# Influence of Cochlear Implantation on Vestibular Function in Children With an Enlarged Vestibular Aqueduct

**DOI:** 10.3389/fneur.2021.663123

**Published:** 2021-04-21

**Authors:** Ruijie Wang, Daogong Zhang, Jianfen Luo, Xiuhua Chao, Jiliang Xu, Xianfeng Liu, Zhaomin Fan, Haibo Wang, Lei Xu

**Affiliations:** Department of Otolaryngology-Head and Neck Surgery, Shandong Provincial ENT Hospital, Cheeloo College of Medicine, Shandong University, Jinan, China

**Keywords:** cochlear implant, vestibular function, EVA, child, vestibular-evoked myogenic potential

## Abstract

**Background:** Cochlear implantation (CI) is becoming increasingly used in the rehabilitation of hearing-impaired patients. Children with an enlarged vestibular aqueduct (EVA) need CI for severe or profound hearing loss, with excellent outcomes in hearing rehabilitation. However, vestibular function influenced by CI in children with EVA has not been clarified. We compared the characteristics of vestibular function in implanted children with EVA and those with a normal cochlea.

**Methods:** In this retrospective case-control study, 16 children with large vestibular aqueduct syndrome (LVAS) and 16 children with a normal cochlea were recruited as the Study and Control Group, respectively. All children (mean age, 10.3 ± 4.4 years) had bilateral profound sensorineural hearing loss (SNHL) and normal pre-operative vestibular functions and underwent unilateral CI. Otolith and canal functions were assessed before CI and 12 months thereafter. Cervical vestibular-evoked myogenic potential (cVEMP), ocular vestibular-evoked myogenic potential (oVEMP), and video head impulse test (vHIT) were evaluated.

**Results:** Full insertion of the electrode array was achieved in all the cases. Preoperatively, no significant differences in parameters in cVEMP between the Study and Control Group were revealed (*p* > 0.05). In pre-operative oVEMP, shorter N1 latencies (*p* = 0.012), shorter P1 latencies (*p* = 0.01), and higher amplitudes (*p* = 0.001) were found in the Study than in the Control Group. The Study Group had shorter P1 latency in cVEMP (*p* = 0.033), and had lower amplitude in oVEMP after implantation (*p* = 0.03). Statistically significant differences were not found in VOR gains of all three semicircular canals before and after surgery (*p* > 0.05). VEMP results revealed that the Control Group had significantly lower deterioration rates after CI (*p* < 0.05). The surgical approach and electrode array had no statistically significant influence on the VEMP results (*p* > 0.05).

**Conclusion:** oVEMP parameters differed between children with EVA and children with a normal cochlea before surgery. Systematic evaluations before and after CI showed that otolith function was affected, but all three semicircular canals functions were essentially undamaged after implantation. In contrast to subjects with a normal cochlea, children with EVA are more likely to preserve their saccular and utricular functions after CI surgery. Possible mechanisms include less pressure-related damage, a reduced effect in terms of the air-bone gap (ABG), or more sensitivity to acoustic stimulation.

## Introduction

Cochlear implantation (CI) is a gold standard therapy for total or severe sensorineural hearing loss (SNHL). In congenitally deaf children, early intervention enables communication, oral language, and cognitive function development. Although studies have shown that CI is effective and safe, the potential effects on vestibular function are of clinical concern (1). Because of the proximity of the cochlea and vestibule, a vestibular impairment may occur after CI, leading to disorders of environmental perception and balance ability (2). Possible reasons include electrode insertion, intraoperative perilymphatic loss, labyrinthitis, endolymphatic hydrops, or electrical stimulation (3).

The vestibular aqueduct is a bony canal in the temporal bone. Arrested development during the fifth week of gestation, before narrowing occurs, results in large vestibular aqueduct syndrome (LVAS) (4). An enlarged vestibular aqueduct (EVA) is the most common inner ear malformation associated with early-onset SNHL, as first described by Mondini (5). As children with EVA become progressively deafer through childhood, they would be ideal candidates for CI (6). Studies have described excellent speech perception outcomes in patients with EVA who had undergone CI (7). However, patients with EVA may have vestibular dysfunctions. According to previous reviews, adverse vestibular signs and symptoms varied from 0 to 100% (8–10). Post-operative vertigo was observed to be increased significantly after CI (11). Some studies have demonstrated that individuals with vestibular impairments showed worse performances in terms of visuospatial ability, attention, executive function, and memory (2). With unilateral or bilateral CI in children with EVA, this risk needs to be carefully taken into account.

Vestibular impairment can be investigated by objective tests. Vestibular-evoked myogenic potential (VEMP) parameters in an EVA patient were recently discussed. A few studies have demonstrated different parameters in VEMPs between patients with EVA and those with a normal cochlea (12–14). The VEMP is used to evaluate the otolith system quantitatively and includes the cervical VEMP (cVEMP) and ocular VEMP (oVEMP). The cVEMP is derived from the saccule and mainly reflects saccular function and inferior vestibular nerve. The oVEMP is derived from the utricle and mainly reflects utricular function and superior vestibular nerve (15). Normal cVEMP and oVEMP responses have been detected in 46.7–100% and 63.5% of children with SNHL compared to 15.6–83% and 45.5% of children with CI (16). However, systematic objective evaluations of peripheral vestibular organ function in children with EVA before CI have seldom been performed.

In the present research, we compared the pre- and post-operative cVEMP, oVEMP, and video head impulse test (vHIT) results in pediatric populations with EVA and a normal cochlea, to gain insight into the vestibular function of these children.

## Methods

### Participants

This retrospective study included 32 children (32 ears), who underwent unilateral CI in our department between November 2016 and November 2019. Across all subjects, the mean age at implantation was 10.3 ± 4.4 years (range: 5–18 years). The indication for CI was based on severe-to-profound bilateral deafness with little benefit from hearing aids. Patients were excluded if they were ≥ 18 years, unable to participate in vestibular assessments, or had undergone previous otologic surgery. Computed tomography (CT) scans of the temporal bone and magnetic resonance imaging (MRI) were performed before surgery. EVA was defined as a vestibular aqueduct diameter > 1.5 mm at the midpoint between the posterior cranial fossa and the vestibule of the inner ear, or an otherwise grossly malformed morphology of the vestibular aqueduct (8). The surgical technique was identical in all patients and was performed by one senior surgeon. All children had normal otolith and canal functions before implantation. All participants underwent vestibular assessments prior to CI and again at 12 months post-surgery. The CIs were all switched off during tests after processor activation.

We divided children into the Study Group and the Control Group. The Study Group included 16 patients (5 females and 11 males; 4 left and 12 right ears). The mean age was 9.2 ± 4.4 years (range, 5–17 years). Pre-operative CT and MRI showed bilateral EVA in all 16 children. There were 13 subjects with congenital deafness and 3 subjects with progressive deafness. In 12 children, the round window (RW) surgical approach was used, and in 4 the extended RW approach was used. A total of 11 children were implanted with a Nucleus CI422, 1 child with a Med-EL FLEX28, 1 child with a Nurotron CS-10A, and 3 children with a Nucleus CI24RECA electrode. In the Control Group, 16 recipients (5 females and 11 males; 5 left and 11 right ears) were included. Pre-operative imaging was normal in all these children. Their mean age was 11.4 ± 4.4 years (range, 5–18 years). There were seven subjects with congenital deafness and nine subjects with progressive deafness. In 10 children, the RW surgical approach was used, and in 6 the extended RW approach was used. A total of seven children were implanted with a Nucleus CI422, one child with a Med-EL FLEX28, two children with a Nurotron CS-10A, and six children with a Nucleus CI24RECA electrode.

### cVEMP

cVEMP was recorded using the Neuro-Audio auditory evoked potential equipment (Neurosoft LTD, Ivanov, Russia). The test was performed with the patients in a seated position. Tone burst stimuli (93 dB nHL and 500 Hz) were delivered via a standard insert earphone (ER-3A). Active recording electrodes with respect to the examination were placed on the region of the upper third of the sternocleidomastoid muscle (SCM) on both sides. The reference electrodes were placed on the upper sternum. The ground electrode was on the nasion. The head was rotated toward the contralateral side of the stimulated ear to achieve tonic contraction of the SCM during recording. The stimulation rate was 5.1 Hz. Bandpass filtering was 30–2,000 Hz. An amplitude ratio over 30% was considered abnormal if the weaker response was from the implanted ear. In the event of bilaterally reduced responses where the asymmetry ratio would be normal, absent responses were considered abnormal (17).

### oVEMP

oVEMP was recorded using the Neuro-Audio auditory evoked potential equipment (Neurosoft LTD, Ivanov, Russia). The electromyographic activity of the extraocular muscle was recorded with the patients in the seated position. Tone burst stimuli (93 dB nHL and 500 Hz) were delivered via a standard insert earphone (ER-3A). The active recording electrodes were placed on the infra-orbital ridge 1 cm below the center of each lower eyelid. The reference electrodes were positioned approximately 1 cm below them. The ground electrode was on the nasion. The results were recorded with eyes open and maximal gaze upwards. The stimulation rate was 5.1 Hz. Bandpass filtering was 1–1,000 Hz. An amplitude ratio over 30% was considered abnormal if the weaker response was from the implanted ear. In the event of bilaterally reduced responses where the asymmetry ratio would be normal, absent responses were considered abnormal (17).

### vHIT

The vHIT device (Ulmer II Evolution, France) was used. The VHIT Ulmer II was equipped with an ultra-sensitive camera that filmed the patient's face from a distance of ~90 cm. The patient was instructed to maintain eye focusing on a stationary object on a screen at about 1 m distance while the examiner manipulated the patient's head with quick and precise head movements. The vestibulo-ocular reflex (VOR) gain was calculated by vHIT software based on head velocity and eye velocity curves. When the head was turned to one side in the plane of the semicircular canal to be tested, the VOR maintained visual fixation. The breaking of visual fixation, shown by a corrective saccade, indicated a respective canal disorder. This test was possible as soon as the child could hold his head steady. The VOR gain of a horizontal semicircular canal (HSC) <0.8 was considered to be abnormal. Both the VOR gain of the superior semicircular canal (SSC) and the posterior semicircular canal (PSC) <0.7 were considered to be abnormal (18).

### Statistical Analyses

Data were analyzed using the Statistical Package for the Social Sciences (SPSS), version 23.0 (SPSS, Inc., Chicago, IL). Statistical comparisons on parameters were performed using the paired-samples test and the independent-samples test as appropriate. The variables in response rates between groups were compared by the chi-square test. The influence factors on the results were analyzed by the chi-square test. Statistical significance was considered at *p* < 0.05.

## Results

**Figure 1:**
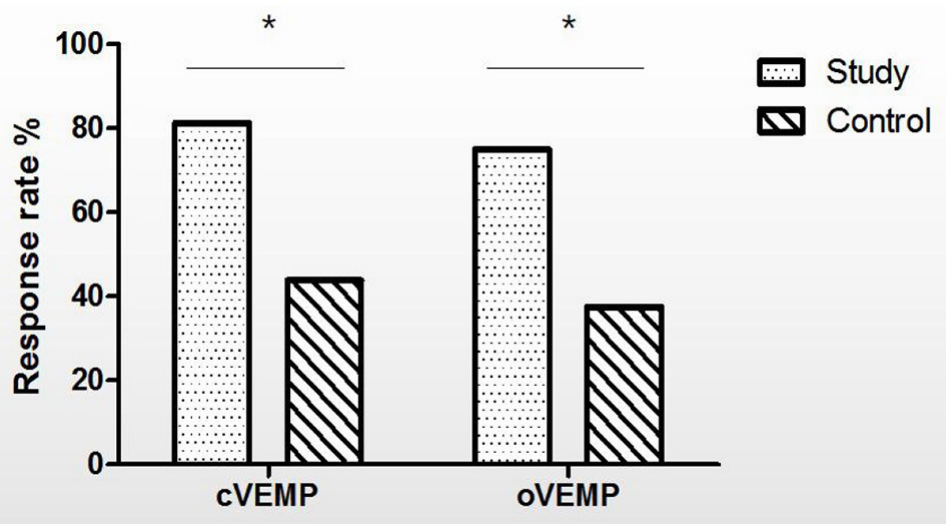
The response rates of cVEMP and oVEMP at postoperative month 12 (%). Chi-square test, the response rate of cVEMP (Study Group vs. Control Group, *p* = 0.028), the response rate of oVEMP (Study Group vs. Control Group, *p* = 0.033), ^*^p < 0.05. cVEMP, cervical vestibular-evoked myogenic potential; oVEMP, ocular vestibular-evoked myogenic potential; Study, Study Group; Control, Control Group.

This study was conducted in two groups of children, who had similar baseline characteristics (Table 1). For all children, each implanted electrode reached full insertion without any resistance or complication. Specific parameters of VEMPs in the Study Group and the Control Group before and after implantation are presented in Table 2. The VOR gains in the vHIT in the Study Group and the Control Group before and after implantation are presented in Table 3. The VEMP response in 32 implanted children and correlation to the electrode and surgical approach after CI are shown in Table 4. The response rates of cVEMP and oVEMP at postoperative month 12 are shown in Figure 1.

**Table 1:** Demographic characteristics of the 32 patients in this study.

**Group**	**Subject number**	**Gender**	**Ear tested**	**Hearing loss**	**Age at implantation (yrs)**	**CT scan**	**Electrode**	**Surgical approach**
Study	S1	M	L	Congenital	6	EVA	CI422	RW
	S2	M	R	Congenital	7	EVA	CI422	RW
	S3	M	L	Congenital	5	MD, EVA	CI422	RW
	S4	M	L	Congenital	8	EVA	CI24RECA	Extended
	S5	M	R	Congenital	5	EVA	CI422	RW
	S6	M	L	Congenital	11	MD, EVA	CS-10A	RW
	S7	F	R	Congenital	5	MD, EVA	CI422	RW
	S8	M	R	Congenital	6	EVA	CI24RECA	Extended
	S9	M	R	Progressive	15	MD, EVA	CI24RECA	Extended
	S10	F	R	Congenital	13	MD, EVA	CI422	RW
	S11	F	R	Progressive	17	MD, EVA	CI422	RW
	S12	F	R	Congenital	6	MD, EVA	FLEX F28	Extended
	S13	M	R	Congenital	7	MD, EVA	CI422	RW
	S14	M	R	Congenital	14	MD, EVA	CI422	RW
	S15	M	R	Progressive	16	EVA	CI422	RW
	S16	F	R	Congenital	6	MD, EVA	CI422	RW
Control	C1	F	R	Progressive	18	Normal	CI24RECA	Extended
	C2	M	L	Progressive	12	Normal	CS-10A	RW
	C3	M	R	Progressive	16	Normal	CS-10A	RW
	C4	F	R	Progressive	12	Normal	CI24RECA	Extended
	C5	M	R	Congenital	5	Normal	CI24RECA	Extended
	C6	M	L	Progressive	18	Normal	CI422	RW
	C7	M	R	Progressive	17	Normal	CI422	RW
	C8	M	R	Congenital	7	Normal	CI422	RW
	C9	F	R	Congenital	6	Normal	CI24RECA	Extended
	C10	M	R	Progressive	13	Normal	CI422	RW
	C11	M	R	Progressive	12	Normal	CI422	RW
	C12	M	L	Progressive	13	Normal	CI422	RW
	C13	F	R	Congenital	11	Normal	CI24RECA	Extended
	C14	M	L	Congenital	10	Normal	CI24RECA	Extended
	C15	M	R	Congenital	7	Normal	FLEX F28	RW
	C16	F	L	Congenital	6	Normal	CI422	RW

*RW, round window; Extended, extended RW; EVA, enlarged vestibular aqueduct; MD, Mondini; M, male; F, female; L, left; R, right*.

**Table 2:** Specific parameters of VEMPs in the Study Group and the Control Group before and after implantation.

**VEMP**	**Group**	**T^▾^**	**P1-pre**	**N1-pre**	**Amplitude-pre**	**P1-post**	**N1-post**	**Amplitude-post**
cVEMP	Study	14	15.38 ± 2.82	23.55 ± 4.48	94.04 ± 51.27	13.66 ± 0.71^*^	25.77 ± 16.54	110.96 ± 60.94
	Control	5	13.85 ± 2.58	21.55 ± 2.59	162.19 ± 122.92	15.38 ± 2.82	21.76 ± 1.59	89.90 ± 43.11
oVEMP	Study	14	14.28 ± 0.96	9.86 ± 0.51	15.18 ± 8.51	14.79 ± 1.33	10.39 ± 1.25	8.16 ± 5.49^*^
	Control	5	15.87 ± 1.42	11.80 ± 2.26	9.04 ± 10.73	15.03 ± 1.48	11.48 ± 1.61	10.50 ± 13.03

*The first positive wave in the cVEMP waveform is P1, and the first negative wave is N1. The first negative wave in the oVEMP waveform is N1, and the first positive wave is P1. P1, ms; N1, ms; Amplitude, μV. T*^▾^*, tested ears of patients with both pre-operative and post-operative present VEMPs; patients with present VEMPs preoperatively and absent VEMPs postoperatively were not included. cVEMP, cervical vestibular-evoked myogenic potential; oVEMP, ocular vestibular-evoked myogenic potential; pre, pre-operation; post, post-operation*;

* *p < 0.05*.

**Table 3:** The VOR gains in the vHIT in the Study Group and the Control Group before and after implantation.

**vHIT**	**Group**	**T**	**SSC-pre**	**HSC-pre**	**PSC-pre**	**SSC-post**	**HSC-post**	**PSC-post**
	Study	16	1.03 ± 0.08	1.02 ± 0.06	0.98 ± 0.09	0.98 ± 0.18	0.90 ± 0.28	0.94 ± 0.13
	Control	16	1.01 ± 0.07	0.98 ± 0.08	0.99 ± 0.10	1.05 ± 0.07	1.01 ± 0.08	1.00 ± 0.09

*Pre, pre-operation; post, post-operation; T, test ears; vHIT, video head impulse test; HSC, horizontal semicircular canal; SSC, superior semicircular canal; PSC, posterior semicircular canal*.

**Table 4:** The VEMP response in 32 implanted children and correlation to electrode and surgical approach after CI.

**Factor**	**cVEMP-normal (n)**	**cVEMP-abnormal (n)**	**oVEMP-normal (n)**	**oVEMP-abnormal (n)**
CI422	12	6	11	7
CI24RECA	4	5	4	5
FLEX 28	1	1	1	1
CS-10A	3	0	2	1
RW	15	7	13	9
Extended RW	5	5	5	5

*Chi-square test. cVEMP, cervical vestibular-evoked myogenic potential; oVEMP, ocular vestibular-evoked myogenic potential; RW, round window; Extended, extended RW; n, number of patient*.

### VEMP Parameters Before Surgery

In the pre-operative cVEMP test, the means of P1 latencies, N1 latencies, and amplitudes of the Study Group (*n* = 16) and Control Group (*n* = 16) were 15.04 ± 2.79 ms vs. 14.24 ± 1.62 ms, 22.86 ± 4.59 ms vs. 21.72 ± 2.08 ms, and 94.99 ± 49.40 μV vs. 88.61 ± 86.82 μV, respectively. The independent-samples test showed that there were no significant differences between the pre-operative parameters of these two groups (*p* > 0.05). In the pre-operative oVEMP test, the N1 latencies, P1 latencies, and amplitudes of the Study Group (*n* = 16) and Control Group (*n* = 16) were 9.92 ± 0.62 ms vs. 11.26 ± 1.68 ms, 14.52 ± 1.21 ms vs. 15.65 ± 1.29 ms, and 13.87 ± 8.71 μV vs. 5.63 ± 6.63 μV, respectively. The independent-samples test showed that N1 latencies (*p* = 0.012) and P1 latencies (*p* = 0.01) were shorter, and amplitudes (*p* = 0.001) were higher in the Study Group than the Control Group.

### Changes in VEMP Parameters Between Pre- and Post-CI

In the Study Group, two children with normal VEMPs before CI had absent VEMPs (cVEMP or oVEMP) postoperatively. Paired-samples test showed that shorter P1 latency in cVEMP (*n* = 14, *p* = 0.033) and lower amplitude in oVEMP (*n* = 14, *p* = 0.03) were found after implantation in the Study Group (Table 2). In the Control Group, 11 children with normal VEMPs before CI had absent VEMPs (cVEMP or oVEMP) postoperatively. The paired-samples test showed that no significant changes in all three parameters (P1, N1, amplitude) after as compared to before surgery (*n* = 5, *p* > 0.05; Table 2).

Six children implanted with the Nucleus CI 422 electrode (RW approach), five children implanted with the Nucleus CI24RECA electrode (extended RW approach), one child implanted with the Nurotron CS-10A electrode (RW approach), and one child implanted with the Med-EL FLEX 28 electrode (RW approach) demonstrated present VEMPs preoperatively and absent postoperatively (cVEMP or oVEMP), and were excluded from analysis of VEMP parameter changes.

### Response Rates of VEMP

In the Study Group, three children had abnormal cVEMP responses and four children had abnormal oVEMP responses after surgery. Two showed decreases in the amplitude of cVEMP and one showed no response, while two showed decreases in the amplitude of oVEMP and two showed no responses. The response rates of cVEMP and oVEMP decreased to 81.25 and 75.00%, respectively, after CI.

In the Control Group, 9 children had abnormal cVEMP responses and 10 had abnormal oVEMP responses after surgery. Two children showed decreased and seven had absent cVEMP responses, while one child had decreased response and nine had absent oVEMP responses. The response rates of cVEMP and oVEMP decreased to 43.75 and 37.50%, respectively, after CI.

After CI, children with abnormal VEMP responses included these 13 children who had present VEMP preoperatively but absent VEMP postoperatively (cVEMP or oVEMP). There were 2 children with EVA and 11 children with a normal cochlea.

The chi-square test showed that the response rate of cVEMP was statistically significantly lower in the Control Group than in the Study Group (*p* = 0.028), and the response rate of oVEMP was statistically significantly lower in the Control Group than in the Study Group after CI surgery (*p* = 0.033) (Figure 1).

### VOR Gains and Response Rates of vHIT

The pre-operative SSC VOR gain was compared between the two groups, but the independent-samples test showed that the difference was not statistically significant (mean gain in the Study Group = 1.03 ± 0.08, mean gain in the Control Group = 1.01 ± 0.07, *p* = 0.402). The pre-operative HSC VOR gain was not statistically significantly different between the two groups (mean gain in the Study Group = 1.02 ± 0.06, mean gain in the Control Group = 0.98 ± 0.08, *p* = 0.08). The pre-operative PSC VOR gain was also not statistically significantly different between the two groups (mean gain in the Study Group = 0.98 ± 0.09, mean gain in the Control Group = 0.99 ± 0.10, *p* = 0.642) (Table 3). The paired-samples test showed that VOR gains in the HSC, SSC, and PSC did not differ differently before and after surgery within groups (*p* > 0.05) (Table 3).

In the Study Group, one child with EVA had post-operative abnormal VOR gains in all three semicircular canals, and one child with EVA had post-operative abnormal VOR gains in the HSC. The response rates of all three semicircular canals were all 100% in the Control Group postoperatively.

### Influence of Surgical Approach and Electrode Array on VEMP Results

The electrode array and surgical approach used had no statistically significant impact on the changes pre- and post-CI in the patients overall (chi-square test, *p* > 0.05) (Table 4).

## Discussion

In this study, we compared the vestibular function characteristics in implanted children with EVA and those with a normal cochlea. We found that oVEMP parameters differed between children with EVA and children with a normal cochlea before surgery. Systematic evaluations before and after CI showed that otolith function was affected, but all three semicircular canals functions were essentially undamaged after implantation. In contrast to normal children, children with EVA were more likely to have preserved saccular and utricular functions after CI.

Cochlear implants are hearing prostheses that bypass defective sensory hair cells in the cochlea, allowing individuals with severe to profound SNHL to regain much of their hearing. Effects of CI on pediatric and adult vestibular receptors were discussed in researches before. Previous studies have shown that most patients experience vertigo symptoms and the canal and otolith function could be damaged after CI (19–23). In a previous study, vHIT revealed that 30% of patients demonstrated a post-operative change in vestibular function (24). However, few studies have investigated the vestibular function in children with CI. Most of these studies analyzed the caloric and cVEMP results in children and showed deteriorated HSC and saccular functions after CI (25–29). A few reports have studied oVEMP and vHIT tests in children with CI (28, 30, 31). It has been suggested that doctors should be aware of potential vestibular dysfunction in LVAS patients (9). Systematic studies of post-CI peripheral vestibular organ function in children with EVA have been rare.

In the present study, before CI, shorter P1 latencies, shorter N1 latencies, and higher amplitudes of oVEMP were found in LVAS children than in children with a normal cochlea. Taylor et al. (13) and Zhou et al. (12) found higher oVEMP amplitudes in patients with LVAS, similar to our present findings. However, another report showed no significant difference in oVEMP parameters in children with EVA (14). Higher cVEMP amplitudes have also been reported in children with EVA (12, 32, 33), which was in contrast to our findings. The reasons for the disparate findings among studies are unknown. The largest cVEMP amplitude in response to tone bursts occurred between 600 and 1,000 Hz, while the largest oVEMP amplitude in response to tone bursts was found at 500–1,000 Hz (34). A recent report demonstrated that cVEMP showed more disparities in parameters. Adult patients had more severe impairment of the vestibular apparatus with aging (14). Different ranges of frequencies are needed at different ages to evoke the best VEMP responses (35). Recently, some studies have shown that the observed modulation of oVEMP responses by increased intracranial pressure (ICP) is primarily due to the effect of an increased intralabyrinthine pressure on the stiffness of the inner ear contents and the middle ear-inner ear junction. Reduction in ICP by lumbar cerebrospinal fluid (CSF) drainage has a systemic effect on VEMP amplitudes. Increasing ICP systematically alters oVEMP in terms of absolute amplitudes and frequency tuning characteristics (36–39). In this report, in terms of differences in oVEMP parameters between children with EVA and those with a normal cochlea, we speculated that the presence of a third window in the inner ear labyrinth might allow for activation of vestibular receptors in LVAS patients (40). LVAS is regarded as a third-window lesion disease: this refers to an additional opening to the inner ear except for the first and second windows. A similar characteristic can be found in other third window diseases, such as SSC or PSC dehiscence (10). The sound energy could be shunted away from the cochlea to the vestibule, making the vestibular system organs more excitable and sensitive, leading to a shorter latency or higher amplitude. In this study, a stronger oVEMP response was demonstrated in children with EVA. This phenomenon implied that the utricular function might be more sensitive to sound in children with EVA than in those with a normal cochlea.

In this report, all children had normal otolith and canal functions before surgery. In LVAS children, the response rates of cVEMP and oVEMP decreased to 81.25 and 75.00% after CI. In children with a normal cochlea, the cVEMP and oVEMP response rates decreased to 43.75 and 37.50%, respectively. We found that otolith function was markedly affected after CI, particularly in children with a normal cochlea. Several studies have described the otolith organs as being the most frequent site of damage (41). Otolith sensors can be susceptible to surgical damage following electrode insertion, drilling, variation of the inner ear environment, or electrical stimulation related to CI. Significantly lower VEMP response rates were found in subjects with a normal cochlea. It seemed that otolith function was relatively less damaged after CI in children with EVA. A series of recent investigations have reported that the pressure within the cochlea may change during the insertion of CI electrodes (42–44). It has also been verified that the vestibular end organs are at risk to be injured by the pressure-related trauma during cochlear implant insertion (45). The pressure energy was confirmed to be propagated from the cochlea to the vestibular labyrinth in the absence of a third window (46). Based on our results, we hypothesized that the pressure change generated during the insertion of electrodes might be released through the EVA or released into the endolymph fluid in patients with EVA. Therefore, children with EVA might eventually protect against vestibular function loss due to cochlear implantation by equalizing the pressure inside the inner ear. However, decreased or absent VEMP responses may not necessarily reflect otolith dysfunctions. Furthermore, previous studies confirmed that the sensitivities to acoustic stimulation of the utricle and semicircular canal can be increased in the presence of a third window (47, 48). Since then, it was speculated that although the impairment of otolith function occurred, children with EVA were more sensitive to acoustic stimulation and had less change in VEMP results, as discussed aforementioned. It has been proposed that the air-bone gap (ABG) might adversely affect the air conduction stimulation (ACS) responses of VEMP (49). A study found that mechanical changes could lead to an ABG, which varied across patients, with an unclear mechanism (50). VEMPs were reported to be present in ears with ABG and LVAS (10). Hence, we considered that CI affected ABG in a different manner in children with EVA. Our data suggested that the post-CI otolith function in children with EVA might be less susceptible to ABG. The mechanism for the different performances between children with EVA and children with a normal cochlea remains unknown and needs further in-depth research in the future.

A shorter P1 latency of cVEMP and lower amplitude of oVEMP were seen in children with EVA in this study. The decrease in oVEMP amplitude was consistent with a previous report of children with SNHL (31). We excluded 13 children with present VEMP preoperatively but absent VEMP postoperatively when comparing the parameters. Different surgical approaches and electrode arrays were used in them. Some studies failed to find a correlation between the post-operative vestibular symptoms and gender, implanted side, age, implant type, and the results of Caloric and VEMP test before (21, 26). The data on the relationship between VEMP response and different influence factors is currently lacking. We analyzed the influence of the surgical approach and electrode array on the changes in VEMP response but found no effect on the changes from pre- to post-operation in this study.

vHIT is a fast, practical, and non-invasive test used to evaluate all three semicircular canals. It uses a more physiological stimulus, testing higher frequencies (> 1 Hz), which is similar to the physiological stimuli of daily life (51). HSC VOR gain observed by vHIT was studied in a previous case report (52). In this study with the aid of vHIT, the VOR gains of all three semicircular canals were not statistically significantly different between groups. The post-operative response rates of all three semicircular canals were 100% in normal children. In children with EVA, there were no statistically significant response rate variations of any of the three semicircular canals from pre- to post-operation. However, HSC functions (two children), SSC function (one child), and PSC function (one child) were damaged in children with EVA after CI. Post-mortem temporal bone studies suggested that CI can cause structural damage to the inner ear, including the posterior labyrinth (53, 54). The HSC function might be easily influenced after surgery, as this is the explored part of the posterior labyrinth. The mechanism involving the function of all three semicircular canals in children is still being studied.

## Limitations

When we compared the changes of parameters in VEMP from pre- to post-CI, we excluded 11 children in the Control Group and 2 children in the Study Group who demonstrated normal VEMP responses preoperatively but absent postoperatively. Therefore, the numbers of children were different between groups. We observed the changes in latency and amplitude in the two groups separately.

## Conclusion

Our research findings further validated the value of VEMP and vHIT tests in the clinical application of vestibular evaluations in children. The utricular function was found to be more sensitive to sound in children with EVA. Although otolith function was affected, the overall damages to all three semicircular canals functions were slight after implantation. In contrast to subjects with a normal cochlea, the otolith sensor function was less seriously affected in children with EVA after CI surgery. Possible mechanisms include less pressure-related damage, less of an effect resulting from ABG, or more sensitivity to acoustic stimulation.

## Data Availability Statement

The raw data supporting the conclusions of this article will be made available by the authors, without undue reservation.

## Ethics Statement

The studies involving human participants were reviewed and approved by the ethics committee of Shandong Provincial ENT Hospital. Written informed consent to participate in this study was provided by the participants' legal guardian/next of kin.

## Author Contributions

LX, HW, RW, and DZ contributed to the conception of the work. JL, XC, and ZF contributed to the experimental design. JX and XL selected data and performed the analysis. All authors contributed to the interpretation of the data and were involved in writing the manuscript.

## Conflict of Interest

The authors declare that the research was conducted in the absence of any commercial or financial relationships that could be construed as a potential conflict of interest.
